# Families between care, education and work: The effects of the pandemic on educational inequalities in Italy and Milan

**DOI:** 10.1111/ejed.12483

**Published:** 2021-10-19

**Authors:** Marta Cordini, Gianluca De Angelis

**Affiliations:** ^1^ Politecnico of Milan Milan Italy; ^2^ Politecnico of Milan and IRES‐ER CGIL Milan Italy

## Abstract

Italy was the first Western country affected by the pandemic. The school closures that followed lasted for a full school semester, including final exams. Italy is already known as a country with a high degree of educational inequality, where reproduction of social disadvantages by social origins is prominent. In such a situation, we hypothesise that a prolonged lockdown and the consequent reliance on remote education have played an important role in exacerbating existing inequalities. Families were forced to take on the full responsibility of educating their children, which has reinforced the role of household resources. The analysis presented in this article draws on results from an online survey of the effects of school closures on educational practices, the analysis focused on responses from parents of primary school students. The survey was carried out in Milan in June 2020 and was disseminated through institutional and informal channels after two months of school closure in Italy. Results were re‐balanced to represent different social classes. The survey explored the relationships between economic and social inequalities on the one hand, and school activity experienced at home during the lockdown on the other. It also explored the involvement of pupils and their parents in school activities. We aimed to capture what educational and organisational resources families were able to mobilise in this situation, and to what extent these are unequally distributed. Our results contribute to an understanding of the impact of family resources on educational chances, identifying resources and how they are distributed through the population. Our findings confirm that the pandemic has exacerbated already existing inequalities.

## 1 INTRODUCTION

School closures in 2020 were a sudden and unexpected event for most countries in Europe. The forced closure of schools has nurtured the debate on the increasing structural inequalities that characterise not only the school itself but also its intake, families and territories. On one hand, the multifaced role of the school has emerged; on the other hand, the flaws of a system that cannot hinder the reproduction of inequalities has been highlighted. Italy is already known as a country with a high degree of educational inequality, where the reproduction of social disadvantages by social origins is so strong that the chances for social mobility of the less advantaged are significantly reduced. This peculiarity of the Italian system has been emphasised by the pandemic, as households and the domestic space are involved in school activities to a greater extent than usual.

The long‐term closure of schools has reinforced the role of household resources. These resources include not only the cultural and social capital of parents, but also the digital equipment, the availability of a private space to study, the extent to which housing is overcrowded, the time at disposal for parents and other family members to assist children in their learning activities.

This article contributes to an understanding of how the socio‐economic background has affected the educational experiences during the pandemic. The analysis is empirically based on the results of an online survey carried out in Milan in June 2020. The survey targeted parents of primary school students and was distributed through institutional and informal channels after two months of school closure. We collected about 4,000 responses. Results were re‐balanced to represent different social classes. The survey explored the relationships between economic and social inequalities on the one hand, and school and/or educational activities carried out at home during the lockdown on the other. Our aim was to capture what educational and organisational resources families were able to mobilise in this situation, and to what extent these are unequally distributed.

## 2 THE CONTEXT OF PANDEMIC AND SCHOOL CLOSURES

On 4 March 2020, the Italian national government established school closures nationally to contain the spread of COVID‐19. On that date, 3,089 cases were reported in Italy, of which 1,820 only in Lombardy. This first decree established the suspension of activities for all grades and levels of education for the next fifteen days, a measure that was renewed by following decrees until the end of the school year. Eventually, schools in Italy reopened in September 2020. Schools and families were unprepared. After the first weeks of disorientation, schools and families started to organise themselves as it became evident that the closures were not temporary. Italy was not an isolated case as most European countries decided to close schools. Nevertheless, Italy was one of the EU countries where closures lasted longer.

The first measures addressing explicitly and directly the reopening of schools were issued at the end of June 2020 in the *Piano Scuola* 2020–2021 (School Plan 2020–2021) and a Technical Report from the Civil Protection (Dipartimento della Protezione Civile, 2020): these documents established that early childcare services were to open 7 September and instruction from primary to upper grades on 14 September. Before 24 June, 2020, when these documents were disseminated, families were in the dark regarding school activities. This delay in organising the re‐opening is connected to several features of the Italian school system ranging from issues regarding school buildings, that are generally dated, to the ageing of the teaching population, one of the oldest in Europe, and the large sizes of classes. In addition, the diversity of the Italian territory made the attempt to release a national protocol applicable to all the school contexts challenging.

The consequences of prolonged school closures go beyond the field of education. Of course, the most evident adverse effect is the interruption in learning—much more harmful for students with disadvantaged backgrounds since schools represent for them the main and sometimes the only context for educational opportunities (Esping‐Andersen, 2008; Sabatinelli, 2016). Also, schools provide especially vulnerable students the benefit of socialisation outside the family context (Bonal & Gonzalez, 2020).

Evidence from the United Kingdom suggests that children from better‐off families spent 30% more time on learning at home than those from poorer families during the lockdown, and their parents reported feeling more able to support them than socio‐economically disadvantaged parents, while students from richer schools had access to more individualised resources (such as online tutoring or chats with teachers; IFS, 2020).

Students who previously received free school meals, those from lower educated and single parent families, and those with immigrant background, devoted significantly less time on schoolwork at home during the COVID‐19 school closures in the UK (Bayrackdar & Guveli, 2020). Even when students have been able to access remote learning, education during school closures seems to have widened the existing attainment gap between students from different socio‐economic backgrounds (Coe et al., 2020).

Secondly, families were, as mentioned before, unprepared for distance learning and home schooling. This unpreparedness can be due to different factors: low educational status of parents, issues negotiating care and work duties, language difficulties for immigrant families, not having a suitable environment for distance learning (i.e., lacking a proper space, devices shared with other family members, poor internet connection etc.) (Adams, 2020). For example, in many OECD countries, fewer than half of rural areas have fixed broadband at sufficient speed (OECD, 2020).

In addition, schools were unprepared and teachers were not trained for distance teaching; also, working hours, duties and expectations were blurry (Di Nunzio et al., 2020). The management of remote teaching was left to schools, resulting in a great heterogeneity in the service provided that has widened the existing inequalities between schools and by consequence between families (Santagati & Barabanti, 2020). In general, school closures have exposed the lack of preparation, training and support that teachers have experienced before and during the pandemic in terms of digital and technological competencies (Trust & Whalen, 2020).

The closures have also affected the ability of parents to work. A significant share of working parents relies on childcare services and schools. In countries such as France, Germany, Italy, the UK and the US, 60% of parents have been unable to find alternative solutions for schools and day‐care centres (UNESCO, 2020). Difficulties in negotiating work, family, home, and education related duties in absence of external services has put families and children in stressful circumstances, and in some cases, the burden has involved also other family members such as grandparents (Daks et al., 2020). School is not only a place for academic learning, but also an arena for development, socialisation, relational life and emotional support, that are all important factors for children's psychological wellbeing and adjustment (Larsen et al., 2021). In addition, school routines support children to have regular bedtimes and physical activities, limiting sedentary behaviours and screen time (Brazendale et al., 2017). Distance education during COVID‐19 restrictions was accompanied by strict social isolation measures: social isolation was associated with loneliness, negative consequences on mental health and other health‐related behaviours in children (Larsen et al., 2021; Loades et al., 2020). For families, school closures have strongly affected the division of labour and the daily routines. The sudden changes to family routines included, for example, the reallocation of household tasks, children and parents spending more time at home, different uses of the available space and a multiplication of functions for the space. These disruptions, in some cases, have led to increasing tensions among household members (Biroli et al., 2020). Yet, for some families the closure of schools has also brought some positive consequences, for instance an increase in family time and the reduction of some stress factors (Bruining et al., 2020). Nevertheless, all research converges in sustaining that the most vulnerable families and children are those who have been affected more negatively by the pandemic.

The literature has highlighted how the socio‐economic conditions have had a significant impact on the seriousness of the loss caused by school closures: children coming from disadvantaged families are more likely to experience several effects beyond learning loss, such as a lack of socialisation and integration, a risk of dropping out of school and long‐term effects on achievement (Doyle, 2020; Larsen et al., 2021). The gap between these students and those coming from highly educated and wealthier families is likely to become wider because of the resources that the latter can mobilise to counteract the lack of schooling (Bonal & Gonzalez, 2020). Highly educated families are, for instance, more willing and likely to provide their children with extra‐curricular activities and to expose them to other cultural and educational opportunities, both in pandemic and not pandemic times.

Literature shows that the factors playing a relevant role in promoting educational attainment are various and intertwined: individual features, family background, type of school—not only related to the teaching quality but also to the capability of involving families—and finally factors related to the combination of these three elements (Barone, 2006; Besozzi & Colombo, 2007; Giovannini & Palmas, 2002; Lareau, 2011; Yang, 2003). School achievement seems to depend strongly on the capital to which students have access: economic, cultural, human and social capital (Bourdieu, 1986; Buchmann, 2002; Coleman, 1988, 1990). Parental involvement in the education of students begins at home with the parents providing a safe and healthy environment, appropriate learning experience, support and a positive attitude about school (Durisic & Bunijevac, 2017). Several studies have highlighted the relevance of parental involvement in affecting educational attainment (Pomerantz et al., 2007) by enhancing the cognitive and intellectual development (Grolnick & Slowiaczek, 1994; Pomerantz et al., 2007). Specifically, parents may provide resources that not only cultivate skills but also motivation with the result that children internalise a good attitude towards school, education and learning. Structured, adult supervised activities at home, for instance, tend to favour children in cognitive tests (Downey, 2002), reading to children provide them higher pre‐reading skills and having a home equipped with a variety of educational objects (such as books, magazines, a computer, a place to study) contributes to the educational attainment. Many of these parental practices are highly correlated with socio‐economic status (Balli et al., 1997).

## 3 EDUCATIONAL INEQUALITIES IN THE ITALIAN SYSTEM

### 3.1 Where the pandemic has hit

The crisis brought on by the pandemic has affected an education system already characterised by considerable inequalities. The distribution of educational opportunities from childcare services through tertiary education over the national territory is starkly unequal and the quality of the service provided significantly varies between northern and southern Italy and also within regions and municipalities. Rural and marginal areas suffer from a lack of education services (Sabatinelli, 2016). The territorial divide is accompanied by gender inequality that affects education opportunities and attainment. For literacy, according to PISA survey data (2018), Italy ranks among OECD countries between the 23rd and 29th places (Luperini & Puccetti, 2020). Italy has managed to reduce the share of students dropping out of school from 20.8% in 2006 to 13.8% in 2016, but remains above the EU average (10.7%).

Considerable heterogeneity has been reported not only among territories, but also among schools, in terms of equipment, choices, digital competencies and media education (Censis, 2020). According to Census data, 84.2% of schools have had to provide students with digital devices. In addition, the ageing school staff (46 years is the average age) has not helped in the acquisition of new digital competencies. As the *Save the Children Report* has highlighted (2020), the efforts to reach students in their home have often been useless because of the housing conditions: 42% of households are overcrowded. In addition, 12.3% of students between six and seventeen years lived in houses not equipped with digital devices, in 2019.

Of those who have digital devices, 57% have to share them with other household members for work or study. Finally, only 30% of students have high digital competencies. As anticipated, The COVID‐19 pandemic in Italy has affected an education system that historically and structurally has reproduced inequalities rather than mitigated them. This reproduction moves along a vertical and a horizontal axis as Triventi has shown in his review of the Italian case (2014). The vertical axis concerns the influence of the origin, meaning the family background, on education achievement and career (Checchi et al., 2006; Schizzerotto & Barone, 2006). The horizontal axis refers instead to the controversial effect of the tracking mechanism: in fact, while the horizontal stratification in Italy is lower than in other countries, still several scholars have highlighted how the probability of attending schools considered more performative and paving the way to higher education (*licei*) is higher among students with highly educated parents, while those coming from households with a lower socio‐economic and educational level are more likely to attend specialised schools (technical and professional schools) (Checchi & Flabbi, 2007; Triventi, 2014). Our conceptual framework builds therefore on existing inequalities in terms of socio‐economic status and educational levels, developing the analysis through the dimensions of *time* and *space*. *Time* implies taking into account a negotiation of work‐division in families, a delicate issue that has been one of the most debated during the lockdown (Aguiar et al., 2021; Lagomarsino et al., 2020). *Space* refers to the converging of all activities (work, school, leisure) into the domestic space, challenging it and highlighting the divide in terms of possibilities of reinventing it (Yamamura & Tsustsui, 2021).

## 4 SURVEY AND SAMPLING METHOD

The use of web‐based survey platforms is no longer a marketing prerogative. On the one hand, online platforms provide more and more complex tools for data collection and extraction with diversified costs. On the other hand, an increasing availability of internet connections, and use of devices, makes it possible to reach wider and more heterogeneous groups. Such flexibility makes survey platforms such as *Google Forms* and *SurveyMonkey* useful research tools. Among the scientific papers indexed in Scoups since 2005, references to *SurveyMonkey* are found in 1,003. More than 460 in the last three years. The pandemic experience has accelerated remote data collection. In the Social Sciences, one of the sectors most interested in interview techniques, such innovation is of great interest. Nevertheless, using web‐based survey tools implies several methodological issues, both in survey planning and in sample selection. While the flexibility of these tools is an advantage, limitations are also significant, and need to be carefully considered.

In our case, timing played a relevant role both for the planning and the sampling. On the one hand, a survey at the end of the schoolyear would have translated an actual experience of distance learning through memories. On the other, the survey represented a chance to give families an opportunity to speak out about their current experiences, something that we understood as worthwhile beyond our own research purposes. This is the reason why we decided to not define the sample ex‐ante but opted for snowball sampling supported by social networks and official communications from the Municipality of Milan, to enlarge the sample as much as possible.

From 28 May to 6 June, 2020, we collected 4,008 questionnaire responses from parents of 5,213 students enrolled in schools of all grade‐levels, inside and outside Milan. We used the school's postcode to exclude respondents living outside Milan, reducing the sample to 3,648 surveys, of which 1,494 were from primary students. The focus on Milan reinforced some sample distortions. First, our data collection campaign missed non‐Italian families. Despite several attempts, only 3.7% of the sample was composed of immigrants. Second, the educational level of our sample is very high. Only 3.2% of respondents had an educational level lower than International Standard Classification of Education (ISCED) level 3—ISCED 3 represented 23.9% of the sample, 55.4% were ISCED 4 or 5, and 17.5% were PhDs or Postdocs. Because of the self‐selection of the sample, this concentration was expected. In order to counterbalance this distortion, we have weighted the answers. We calculated an index of educational level for both parents. Drawing on descriptions of student family background in the latest national standardised INVALSI (Italian national institute for the evaluation of the national education and training system) examinations (language and math) for primary classes in Milan we obtain the following weights: 3.6 for low educational level (composed by household with educational level not higher than ISCED 2), 1.45 for middle‐low (not higher than ISCED 4), 0.84 for middle‐high (at least one member with ISCED 5) and 0.65 for high educational level (both members with ISCED 5 or higher). By this step, we reduced the sample that we have analysed to 1,453 students enrolled in primary, from 1,235 families.

To represent the occupations in households we created a professional status index as a proxy of the socio‐economic status, alongside parental education levels. The professional status index is based on the occupational levels of household members and on their economic means: our sample is composed of 31.2% families with a high professional status, 39.7% medium, and 29.1% with a low status. The high professional status group includes employed in high level management and white collars, freelance or professionals with a family income starting from 4,000 euros per month. Average professional status group collects households composed by clerks, operative workers, freelance and professionals with a monthly income between 2,000 and 4,000 euros. Finally, the lower professional status group includes families composed by only one employed (not in apical positions) or two members having a precarious job or belonging to the working class. In this case the income is not higher than 2,000 euros per month.

## 5 THE IMPACT ON FAMILIES

### 5.1 Changes in working schedules and financial resources

The closure of schools has had an immediate effect on the managing of time and space in households. The domestic space has been invaded by activities and functions that were once a prerogative of work‐ and leisure areas, schools, or public spaces. Working, learning, teaching, educational and creative activities in a wider sense, leisure activities were all concentrated in the same space and performed by different household members. Research has shown contradicting results on whether the pandemic increased or decreased the hours worked from home (Deole et al., 2021; Lee & Tiptoe, 2020). Nevertheless, Deole and colleagues (2021) found that for parents, the increased childcare responsibilities in the aftermath of school closure reduced their productivity in working from home and increased weekly working hours. Effects on parents' work can be seen in three dimensions: (1) time dedicated to work, (2) workload, and (3) income variation. More than half of our sample did not suffer an income shock (57.9%). Nevertheless, the share of respondents who did is alarming: 29.1% of respondents and 27.8% of their partners have seen their income decrease; for 16.3% of respondents, and for 13.2% of their partners, have entirely lost their income. A quarter of the population have worked more hours than before and more than one third of respondents (39.1%), and for almost a third their partners (29.2%), have suffered from an increase in the workload. This data indicates that an important share of the population has experienced serious changes in their working routines in terms of time, load and income.

The parental education level and professional status have had a significant impact on the way that the work sphere has been affected during the lockdown. Those with a low education level have been the most affected by a reduction of work; this was experienced by more than 60% of the respondents with a low education level. In most of the cases (40.5%) the reduction or the loss of all working hours have concerned each employed member of the household. This circumstance occurred also among those with a low professional status (64.3%). The increase in work hours is instead more prominent when the professional status and the education level are higher: 18.30% of highly educated households have worked more hours during the lockdown compared to the 8.9% among those with a medium‐low and low education level.

**FIGURE 1:**
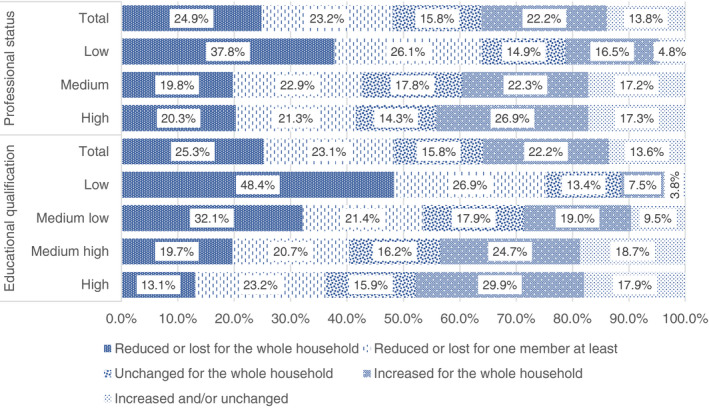
Changes in workload per educational level and professional. *Source*: Figure constructed by authors using questionnaire data [Colour figure can be viewed at wileyonlinelibrary.com]

Also the workload can be significant in reflecting how the balance between family and work duties have been affected by the lockdown (Figure 1). According to our respondents, a high education level was associated with an increase in the workload for almost 30% of respondents, while it affected only the 7.5% of those with low education. On the contrary, a low level of education was associated with a reduced workload for one member in almost half of the cases, and for two household members in 26.9% of the cases. Professional status seemed to have a slightly less strong impact, even if noticeable: those with a low professional status, in fact, have worked less in terms of workload in 63.9% of cases (in 37.8% of these cases this condition has affected only one household member).

These data show a sample divided into two groups: (1) those who have had an increase of hours and energy dedicated to work; and (2) a group who has worked less and has seen the workload diminishing. The first situation tends to feature significantly more households with a high education level and a high professional status, while the second is more likely to be a prerogative of the households with low education level and professional status. Data can also be read in terms of the availability of time to be dedicated to other activities than work, i.e., care or help in educational activities or in general time to be spent with other household members. Accordingly, parents with higher education qualifications and professional status may have faced a struggle in negotiating tasks because of the increased time and energy dedicated to work. In contrast, the part of the sample with lower educational and professional status may have had plenty of time because of the reduction in workload and hours. These conditions are strictly related, of course, to the possibility for some kind of occupation to be carried out at home. As our data suggest and other research has shown (Felstead & Jewson, 2002), high‐skilled, professional and scientific occupations have been more easily performed at home, while low‐service and most of blue‐collar occupations have been abruptly stopped during the lockdown, leading to layoffs or even unemployment. An increased availability of time can often imply a reduction of material resources. A reduction or loss of income concerns more than half of our population. In 40% of these cases income was reduced only for one member. Whilst a reduction or loss of income concerns also a relevant slice of those with a medium‐low qualification (62%), this impact is less serious among those with a medium‐high (44.5%) or a high qualification (38.9%). The situation was similar for professional status.

Our sample appears polarised between households having at their disposal more time but suffering from income loss—that means a reduction also of resources to invest in educational activities or hired care—and households having experienced an increase in the amount of time and energy spent working, but with an unchanged situation in terms of income. The population with lower educational levels and lower professional status have found themselves in a peculiar situation: having more time at disposal to take care of their children's education with less financial resources and with all the consequences of an income shock: financial stress, health related issues, housing hardship and the like. In contrast, the most educated and high‐skilled population, occupying also managerial and high‐status roles, have seen their work burden increase but their financial resources unchanged.

## 6 SCHOOL AT HOME

### 6.1 Negotiating education

The support needed for school activities in some cases (41%) became more problematic when the end of the lockdown was not accompanied by a re‐opening of schools. Families have been sometimes forced to look for support among other adults such as relatives or paid workers. While 45% took on the burden of supporting their children in school activities, almost 20% involved grandparents, 9.4% other family members, and 6.9% baby‐sitters. Of low professional status households 21%, and a quarter of medium professional status households have appealed to grandparents. Among high professional status households this percentage is lower (18%). Low and medium professional status households asked for help from other family's members—9.9% and 7.5% respectively. The comparative percentage for high professional status households was only 2.2%. Finally, we can notice that the externalisation of care is considerably more common among families with high professional status (17.7%) compared to 9.9% in medium professional status families and 2.6% in low professional status families. Remarkably, in all the categories, almost a third of families did not request help from anybody.

**FIGURE 2:**
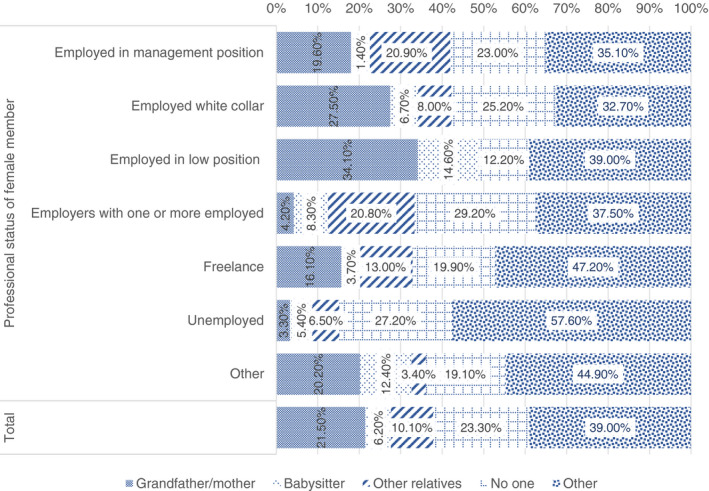
Support by mothers' occupation once the lockdown ended and schools reopened. *Source*: Figure constructed by authors using questionnaire data [Colour figure can be viewed at wileyonlinelibrary.com]

Since the impact on women in terms of increasing domestic and care work has been higher than on men (Bonacini et al., 2021; Cuesta & Pico, 2020), we have tried to look at how the mothers' occupation has affected the kind of support parents have chosen (Figure 2). Expanding this analysis to the work typologies of mothers we can notice that the involvement of relatives, especially grandparents, in caring and school duties is more relevant among blue‐ (34.1%) and white‐collar employees (27.5%), while 20% of entrepreneurs hired a baby‐sitter, and 13% of the freelancers. More than half of the unemployed did not use any support or help and this share decreases by other circumstances, but it is for all respondents higher than 30%.

Matching these data with those about the effects of lockdown on work hours, workload and income, we can understand other features regarding this polarisation. As high professional status professions are the ones more likely to be performed from home, the issue of task negotiation concerned mostly this share of the sample, which is also the one who has more resources to externalise childcare. The other end of the continuum is represented by blue‐collar and low‐skilled workers that have seen a reduction or a loss of work hours and/or income, with a parallel increase in time. But once the restriction measures have been softened some of these workers have returned to work while the schools remained closed. This combination has put these workers in front of negotiation issues that have been mostly solved through unpaid solutions. Another share of this group has not returned to work facing serious financial issues that are likely to have long‐term effects.

## 7 HOUSEHOLD RESOURCES IN TERMS OF HOMES AND DEVICES

**FIGURE 3:**
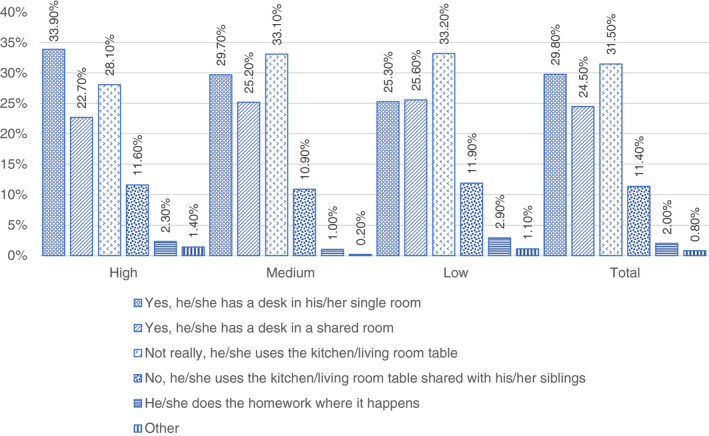
Spaces at home per professional status. *Source*: Figure constructed by authors using questionnaire data [Colour figure can be viewed at wileyonlinelibrary.com]

The time and financial resources of households have been abruptly called on as schools entered domestic spaces in an unprecedented way. The blurring of the borders between school and domestic space has contributed to an increase in inequalities because it has highlighted the difference in household resources. Figure 3 shows the conditions of shared domestic spaces by professional status.

**FIGURE 4:**
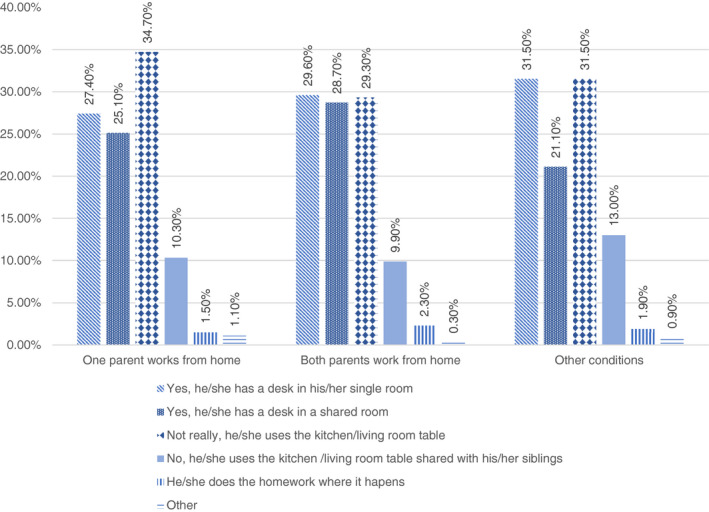
Availability of domestic space to study in households working from home. *Source*: Figure constructed by authors using questionnaire data [Colour figure can be viewed at wileyonlinelibrary.com]

More than a third of children in the sample attended online classes or did homework on a table in the kitchen or the living room (31.5%). Another third attend classes in a single room (29.8%) and almost one half shared a room (24.5%). If we consider professional condition, the availability of a desk in a single room (33.9%) was more common among high professional status households compared to medium (29.7%) and low (25.3%). The availability of space to study and/or work evidently become an issue when several members work or study at home. For this reason, we have considered the availability of space among those households in which all the members or at least one work from home (Figure 4).

As we can see, one third of the children having just one parent working from home tended to use a table placed in the kitchen or the living room (34.7%) and only 10% shared this table with siblings. More than a quarter have a desk in a single room (27.4%) and another quarter (25.1%) in a shared room. Children with both parents working from home were almost equally distributed among those having a desk in a single room (29.6%) and in a shared room (28.7%) or using a table in the kitchen or living room (29.3%). The share of the sample not having a dedicated space even if shared is irrelevant. This indicates that children having both parents or only one parent working from home have spent this period in fairly decent circumstances with a space at their disposal for online classes and homework. This is also due to the socio‐economic profile of those parents who have worked from home that overlaps mostly with those households having a high professional status, as already mentioned.

Only 8.28% of households with a low professional status had both parents working from home and only one third had one parent working from home. In contrast, in the case of medium and high professional status, we have only one third not working from home. The possibility of accessing online classes and other activities is also linked to the availability of digital devices and an internet connection.

**FIGURE 5:**
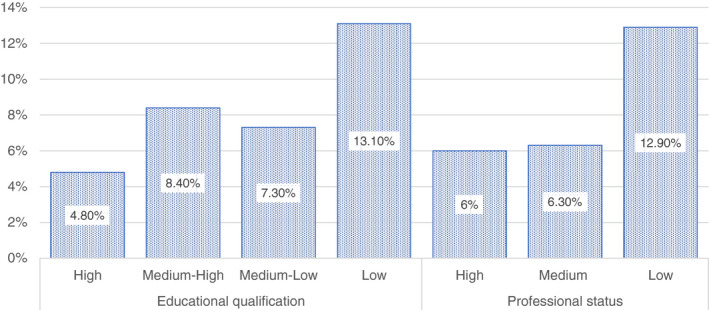
Households without internet connection by educational level and professional position. *Source*: Figure constructed by authors using questionnaire data [Colour figure can be viewed at wileyonlinelibrary.com]

Our sample was in general well‐equipped in terms of internet connection, with more than the 75% of households having a fibre‐optic connection and more than the 16% an ADSL connection. Only 8% were poorly equipped: 6.3% had only mobile phone data and 1.3% had no connection at all. Half of this portion of respondents reacted to the lockdown by installing data on their mobile phone, 17% installed a fibre‐optic or ADLS connection in their house and about one third has remained with no connection. As Figure 5 shows, the concentration of respondents with no connection is among households with low educational level and low professional status.

**FIGURE 6:**
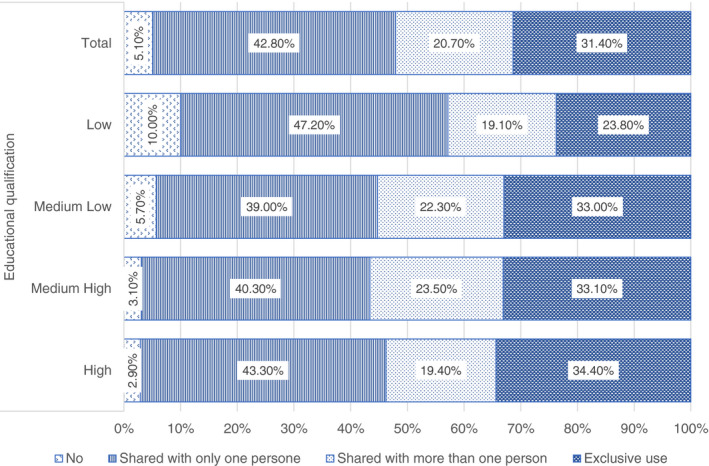
Availability of digital devices at home by educational qualification. *Source*: Figure constructed by authors using questionnaire data [Colour figure can be viewed at wileyonlinelibrary.com]

Having a good connection is not sufficient for assuring a quality online learning, since a proper device is also needed. More than the 40% declare that their child shares the device (laptop or PC) with another family's member and the 20% with more than another member. One third of the children has instead a self‐dedicated device. Considering the educational level (Figure 4), the only relevant difference concerns the respondents with low qualifications: among those the share of child sharing the device with one family's member increases (47.2%), while the share of those who have a self‐dedicated device is lower (23.8%) (Figure 6).

## 8 PARENTAL ENGAGEMENT AND LEISURE ACTIVITIES

The closure of schools alongside with the closure of extra‐curricular activities has challenged parents not only in assisting their children in school‐related activities, but also to fill a void left by other activities such as sport, cultural and social activities. The relevance of these activities is widely acknowledged in supporting cognitive development and achievement in education (La Belle, 1982). Research has showed that the involvement of parents and the quality of learning at home improve academic outcomes (Balli et al., 1997; Downey, 2002; Grolnick & Slowiaczek, 1994).

**FIGURE 7:**
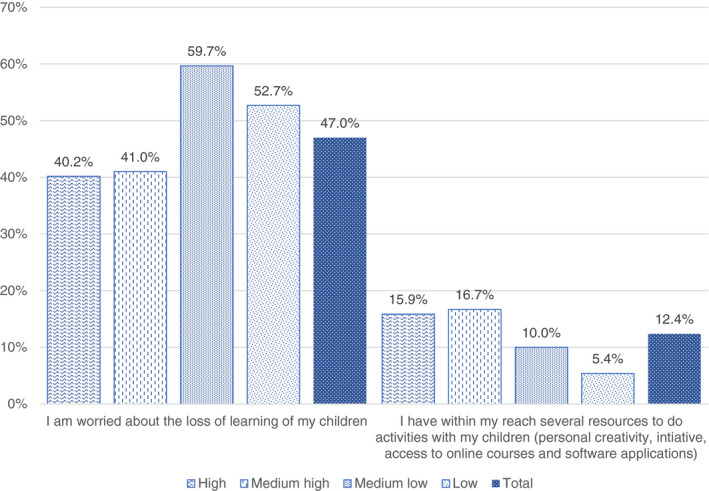
Parents' opinions by educational level. *Source*: Figure constructed by authors using questionnaire data [Colour figure can be viewed at wileyonlinelibrary.com]

Before exploring which activities have been suggested for children in addition to school assignments, Figure 7 shows parents' worries about the loss of learning and the resources they have at disposal (classified as cultural and human resources) to fill or at least diminish this gap. Considering the educational level seems the most adequate proxy to represent the socio‐economic and cultural background. The worry about the loss of learning is more common among households with medium‐low and low levels of education (more than half in both cases) compared to 40% among high and medium‐high professional status families. This can be understood in light of data that 5.4% of those with a low‐level of education and 10% of those with a medium‐low level of education, compared to more than 15% of those with a medium‐high level and high level of education, report having the resources needed. It is also relevant that for all respondents, regardless of educational level and professional status, only 12% of parents perceived that they had the resources to do activities with their children in order to compensate the absence of schools and organised extra‐curricular activities.

**FIGURE 8:**
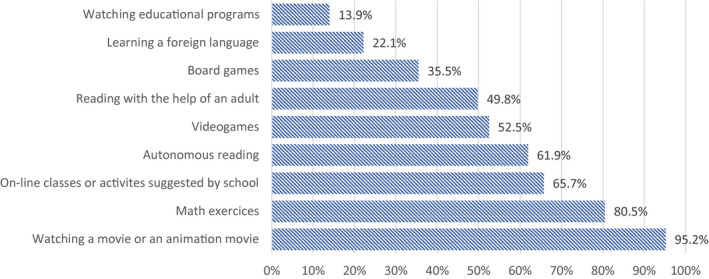
Activities carried out daily or more than once a week in all households. *Source*: Figure constructed by authors using questionnaire data [Colour figure can be viewed at wileyonlinelibrary.com]

Parents were asked about the frequency of certain activities during school closure. Figure 8 shows activities carried out daily or more than once a week for all of the sample. The more common activity was watching a movie (95.2%), followed by doing math exercises (80.5%), here used as a proxy for doing homework. More than half of the sample has been engaged daily or more than once a week in on‐line classes or activities promoted by schools and in reading by themselves. Less common was learning a foreign language (22.15%) and watching educational programs (13.9%).

Our findings indicate the following background has influenced the kind of activities that were carried out frequently by families. The professional status seems to affect which activities are frequently carried out by children to a lesser extent than the educational level of households. The only three activities that seemed strongly affected by professional status were the online classes proposed by schools, watching movies or animation movies and playing board games. Children in higher professional status households tend to carry out these activities more frequently than others.

**FIGURE 9:**
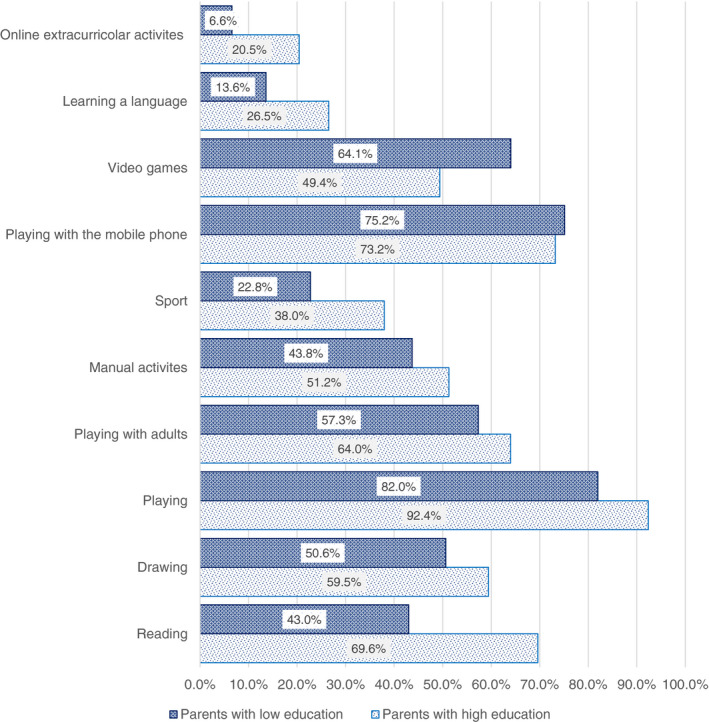
Activities carried out daily or more than once a week per educational qualifications. *Source*: Figure constructed by authors using questionnaire data [Colour figure can be viewed at wileyonlinelibrary.com]

A much stronger correlation was found between the activities in which children have been engaged during the lockdown and the educational level of parents. Out of the activities illustrated in Figure 8, some activities that have been carried out more frequently in highly educated households, such as reading, practicing some sports and learning a foreign language; while other activities, such as video games and playing on the mobile phone are carried out more frequently by lower educated households. Specifically, online extra‐curricular activities seem to be a prerogative of a higher share (20.5%) of highly educated parents (compared to 6.6% and 12.3% of parents with a low‐level education and high‐medium level education respectively). Playing videogames is the activity in which a larger share of children of parents with a low‐level of education engaged (64.1%) if compared to other groups (between 47% and 49%) (Figure 9).

Beyond a discussion on the activities themselves and their impact in terms of cognitive development or education achievement, it is interesting to note how the parents’ education level is so strongly correlated with the type and frequency of activities while the professional status is not. In other terms, we can argue that the education level of households tends to affect the preferences independently from the professional status. This seems to indicate that the level of education is much more determinant in defining the type of engagement of parents and children in certain type of activities, that, in the absence of the education institution are likely to fill the learning gap, or at least make this gap smaller.

## 9 CONCLUSIONS

Research has shown that closure of schools during the summer period increases educational inequalities between socially advantaged and disadvantaged children (Alegre, 2016). Although online classes were organised during the 2020 COVID‐19 pandemic school closures, even when students have been able to access online classes, remote learning seems to have widened the existing attainment gap between students from different socio‐economic backgrounds (Coe et al., 2020). Our survey provides some insights about the factors contributing to this gap, by looking at which resources have been mobilised by families with different socio‐economic profiles.

Clearly, the pandemic polarised households between those that gained time and lost work (in terms of hours, but also in terms of income) and those families who experienced the opposite condition. These latter have faced issues of negotiating work tasks and childcare, the wealthier population with recourse to paid professionals such as baby‐sitters. The former groups have had at disposal plenty of time but less resources, both economically (because of layoffs or unemployment) and culturally (because this situation was prevalent among the share of our sample with a low level of education). In addition, while the recourse to paid solutions could have been dismissed, at least partially, once school reopened, income or work changes can lead to long‐term effects such as unemployment, precariousness or health‐related issues (such as depression) affecting the whole household, children included.

Our respondents were quite pessimistic and negative about their resources to engage their children, regardless of the socio‐economic profile. If we look exclusively at the level of education, leaving the professional status aside, we can notice that a comparatively higher level of education is associated with more frequent engagement in activities contributing to cognitive and educational development, such as reading or learning a language. This strong relevance of educational level seems to imply that while economic resources obviously count, for fostering educational change, it is the education level of households that has made some significant difference during this pandemic in the attempt of filling the void left by school and extra‐curricular activities.
